# From participatory design to clinical routine: new concepts for visualizing patient-reported outcomes in breast cancer care

**DOI:** 10.1186/s41687-026-01008-1

**Published:** 2026-01-27

**Authors:** Lea Doppelbauer, Maria Margarete Karsten, Anna Tatzber, Laura Hatzler, Pimrapat Gebert, Jasper Brands, Rosanne Andriessen, Therese Pross

**Affiliations:** 1 Department of Gynecology with Breast Center, Charité –Universitätsmedizin Berlin, Corporate Member of Freie Universität Berlin and Humboldt Universität zu Berlin, Berlin, 10117 Germany; 2 Department of Urology, Institute of Sexology and Sexual Medicine, Charité—Universitätsmedizin Berlin, Corporate Member of Freie Universität Berlin and Humboldt Universität zu Berlin, 10117 Berlin, Germany; 3 Institute of Biometry and Clinical Epidemiology, Charité– Universitätsmedizin Berlin, Corporate Member of Freie Universität Berlin and Humboldt Universität zu Berlin, 10117 Berlin, Germany; 4 Panton bv, Muntengang 1, Deventer, 7411GD Netherlands

**Keywords:** Patient-reported outcomes, Breast cancer, Data visualization, Qualitative data, Participatory design, Health communication, Patient-centered care, Health literacy

## Abstract

**Background:**

Patient-reported outcomes (PROs) improve cancer care by enabling structured self-assessment of disease-related symptoms, overall functioning, and health-related quality of life. Despite proven benefits, routine use is still limited, in part due to suboptimal data visualization.

**Methodology:**

In a sub-study of the multicenter PRO B trial (52 centers, 924 patients with metastatic breast cancer), an interdisciplinary team of clinicians, researchers, patient representatives, and service designers co-developed user-centered PRO visualizations. Drawing on a literature review, an analysis of existing tools, and iterative mock-up development, the team generated initial design concepts. These concepts were then evaluated in semi-structured interviews with eight patients and five providers to assess clarity, emotional impact, usability, and potential for implementation. The interview data were analyzed thematically using a mixed deductive-inductive approach.

**Results:**

Eight key themes emerged: axis labeling, color schemes, background lines, alert representation, reference data, missing data, format/additional information, and practical application. Preferences included clear labels, consistent directionality, and simple alert markers. Color views diverged: traffic-light schemes were intuitive to some, but evoked negative reactions; blue gradients were perceived as neutral. Reference data aided context for some but discouraged others, supporting optional display. Missing values required consistent marking. Patients preferred access via both paper and customizable digital tools.

**Conclusions:**

Co-designed, user-centered visualizations enhance PRO interpretability, emotional acceptability, and clinical utility. Implementing these recommendations can strengthen communication, support shared decision-making, and promote patient-centered cancer care.

## Abstract (unstructured)

Patient-reported outcomes (PROs) are questionnaires that allow patients to share how they feel, what symptoms they experience, and how their illness affects daily life. These reports can help doctors and patients make better decisions together. However, the way PRO results are currently presented is often confusing and makes it harder for patients and doctors to use them in everyday care. In this study, we worked with patients, doctors, and designers to create clearer and more user-friendly ways of showing PRO results for people with breast cancer. We tested different design options by showing examples to patients and health professionals and asking for their opinions. We found that people wanted clear labels, simple symbols for warnings, and consistent ways to show missing information. Some liked color schemes such as traffic-light colors, while others preferred more neutral designs. Both patients and doctors thought it was important to have results available in both paper and digital formats. Our findings provide practical recommendations to make PRO results easier to understand and more useful in cancer care.

## Background

Patient-reported outcomes (PROs) have become an essential component of modern cancer care. They allow patients to assess their own health status, symptoms, and health-related quality of life (HRQoL) in a structured way. This information adds valuable insights to clinical assessments and helps ensure that treatment decisions reflect not only medical indicators but also the lived experiences and individual preferences of patients [1]. Previous research has shown that regular symptom monitoring using PROs can lead to earlier detection of side effects, more timely interventions, and better overall outcomes [2, 3]. PROs also capture information beyond clinical tests, such as fatigue, emotional burden, and functional limitations. This can be particularly useful for tailoring therapies and identifying when changes in treatment are needed [4]. In addition, PROs support long-term care planning by making it possible to monitor disease progression and the impact of treatment over time [5].

Despite their well-documented benefits, PROs remain underused in daily clinical practice [6–8]. One major barrier is how PRO outcomes are presented [9–11]. Patients and healthcare providers often receive raw scores or tables, which are difficult to interpret, especially for individuals with limited health literacy [12, 13]. For PROs to be clinically useful, their presentation must support rapid, accurate, and intuitive interpretation.

Evidence from health communication, cognitive psychology, and information design shows that visualizations can improve comprehension of complex health information, facilitate the recognition of patterns and trends over time, and support clinical decision-making, especially in demanding care settings [14–18].

However, effective PRO visualization is challenging, as designs must balance clinical accuracy with emotional sensitivity, clarity, and adaptability to diverse user needs. Co-development with patients has been shown to improve usability and acceptance of PRO displays, yet remains insufficiently implemented in routine care tools [19–21].

This study addresses this gap by applying a participatory design approach to develop user-centered PRO visualizations in collaboration with patients and healthcare providers. Embedded within the multicenter PRO B trial [22], which investigated alert-based PRO monitoring in metastatic breast cancer, this study leverages a real-world clinical setting to generate transferable design recommendations for making PRO data more interpretable, actionable, and meaningful in patient-centered oncology care.

## Methods

**Fig. 1:**
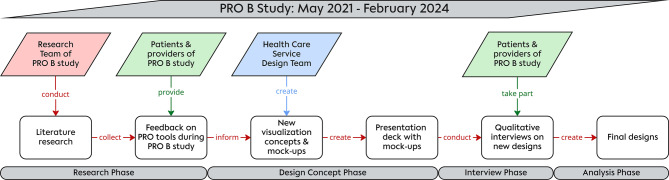
Summary of the research and co-design process indicating the different project stages and parties involved per stage

To ensure that the developed visualizations were grounded in the real-world needs and experiences of users, we followed a multi-step, participatory design process that combined evidence synthesis, interdisciplinary collaboration, and qualitative user feedback. Figure 1 gives a visual summary of the research process.

### PRO B study

The multicentric PRO B^1^ study designed, implemented, and tested an alert-based PRO monitoring system in 52 breast cancer centers across Germany [22, 23]. Centers reflected routine oncology practice in Germany, ranging from large tertiary cancer centers to small rural community clinics. Between May 2021 and February 2024, 924 patients with metastatic breast cancer participated and were randomized into control or intervention group (1:1). Eligible participants were adult patients with metastatic breast cancer who were able to complete electronic PRO assessments and provided informed consent. The PRO B study examined whether systematic ePRO tracking can enhance HRQoL, extend survival, and reduce emergency visits and hospitalizations. Patients in the intervention group answered weekly PRO surveys consisting of a questionnaire based on EORTC CAT Core short forms^2^ (around 50 questions) in an app on their smartphones. Clinicians could view the PRO data in a web-based survey system (PRO tool) and show it to patients during appointments. PRO data were presented in a simple line plot not following an elaborate design concept. In the event of worsening PRO scores, the system automatically sent an email-alert to the patient’s care team. The care team was instructed to contact the patient within 48 hours via phone call to assess their health status and offer support, if necessary. Patients could not view their own PRO results and were not informed if their answers triggered an alert.

### Research

As a first step, an extensive literature review and a review of existing PRO collection tools and their visualizations served as a starting point for new PRO visualization design ideas. We searched PubMed, Embase, Web of Science, and Google Scholar using combinations of the following MeSH terms and keywords: *patient-reported outcome measures, (metastatic) breast cancer, data visualization, data display, user-centered design, clinical practice, patient-provider communication*, *patient education* and *patient-centered care*. Studies were included if they addressed PRO collection or visualization, user-centered design of health tools, or patient communication in oncology settings. Non-English publications and studies without full-text availability were excluded. The search was conducted in January 2024. Additionally, we consolidated feedback from patients and providers in the PRO B study on visualized PRO data used during the trial.

### Design concepts

Building on the insights from the initial research phase, we developed new concepts for visualizing PRO data using a participatory, user-centered design approach.

The design process was conducted in collaboration between the study team (including two clinically active physicians, two healthcare researchers, one study nurse), two patient representatives and a specialized healthcare service design team. This approach aimed to ensure that the visualizations addressed real-world clinical needs, usability, and emotional resonance [24, 25].

First, findings were synthesized and discussed in the team. Based on this, the design team proposed preliminary visualization concepts, which again were reviewed by the full team. Finalized mock-ups were then created by the design team and compiled into a visual presentation deck that formed the basis for subsequent qualitative interviews with patients and providers participating in the PRO B study (see Fig. 1). Graphics were created using Adobe Illustrator.

### Qualitative interviews

Patients and providers for the qualitative interviews were recruited via snowball technique. The main contact partner at four study centers to invite 3–5 patients from both PRO B study groups and 1–2 team members who were willing to participate in an interview. All patients had previously completed routine longitudinal PRO assessments as part of the main study. No additional inclusion criteria were applied beyond those used in the PRO B study [22].

Semi-structured interviews (20–30 minutes each) were conducted by LD (female) in April and May 2024. LD has several years of experience in oncology-related clinical research and qualitative methods, including qualitative interviews with patients and healthcare professionals. She is a trained clinical linguist and has received formal training in the conduction and analysis of semi-structured interviews as part of her academic background. There was no contact between the respondents and LD prior to the interviews, and no dependency existed following their completion. An interview guide (Online Resource 1), including a short introduction of the study’s aim, was used, which was developed through two rounds of feedback involving the study group, two patient advocates and the design team. Only the participant and the researcher were present during the interviews. No field notes were taken. The interviews focused on evaluating patient-centeredness, comprehensibility for both professionals and lay persons, as well as preferences and recommendations for potential implementation in routine clinical practice. Written informed consent was obtained separately for recording, transcription, and use of the data in this sub study. The interviews were transcribed verbatim by research assistants under the guidance of LD. None of the participants commented or corrected their transcript or requested a printed version of it.

### Analysis

Qualitative data were analyzed using thematic analysis following a mixed deductive-inductive process according to Kuckartz [26]. The main thematic categories were derived deductively guided by the most relevant design aspects to be considered when designing graphs (e.g. labels, colors, directionality, see Table 1) as well as by known necessary dimensions for symptom monitoring (e.g. alert representation). Within these, sub-categories were established inductively. Coding was conducted independently by two researchers (LD and TP), with discrepancies resolved through discussion. No software was used to analyze and manage the data. Data saturation was evaluated through iterative discussions between LD and TP and by monitoring the emergence of new themes during coding. Code saturation was considered reached when no new codes or thematic sub-categories emerged from subsequent interviews, consistent with established qualitative research guidelines [34, 35].

**Table 1:** Results of the literature review on relevant design aspects of health data visualizations

Category	Finding	Reference
Chart type	A wide range of studies suggest that both clinicians and patients prefer visual over tabular formats for interpreting PRO data. Bar and line graphs are consistently favored for their clarity, intuitiveness, and ability to show changes over time.	Breidenbach et al., 2021 [7]; McNair et al., 2010 [9]; Fischer et al., 2020 [27]; Albers et al., 2022 [11]
	Simple line graphs are rated highest for ease-of-understanding and usefulness by both patients and clinicians.	Brundage et al., 2015 [10]
	In contrast, text-based formats are least preferred by patients due to complexity, although they are less prone to misinterpretation.	Fischer et al., 2020 [27]; Gilbert et al., 2015 [28]
Colors	Color can improve interpretability: traffic-light systems and shading (e.g., green = normal, red = concerning) help highlight relevant findings.	Breidenbach et al., 2021 [7]; Snyder et al., 2017 [29]
	Colors can trigger discomfort due to negative connotations.	Smith et al., 2016 [30]
	Formats with too many colors are perceived as overwhelming, and color use should remain interpretable in grayscale.	Snyder et al., 2017 [29]; Snyder et al., 2019 [31]
	While some users appreciate shading as a quick visual guide, others found it confusing and ambiguous.	Smith et al., 2016 [30]; Fischer et al., 2020 [27]
Cut-off points	Visual indicators like red circles and threshold lines are preferred over shading for flagging concerning results, aiding quick recognition.	Smith et al., 2016 [30]; Snyder et al., 2017
	Experts warn that cut-offs might be misleading if not well justified.	Breidenbach et al., 2021 [7]
	Clinicians value additional statistical indicators (e.g., significance stars), whereas patients often misunderstood or overlooked them.	Smith et al., 2016 [30]
	Emphasizing “important” differences rather than technical significance is recommended.	Snyder et al., 2017 [29]
Item order and structure	A clear, thematically structured layout of PRO results is generally preferred.	Breidenbach et al., 2021 [7]
	Most participants support listing all scores rather than only abnormal ones for a more complete picture. Alternative suggestions include prioritizing the most concerning scores at the top.	Albers et al. 2022 [11]
Directionality	Inconsistent directionality (e.g., high = good for functioning, high = bad for symptoms) hinder interpretation. Both patients and clinicians find mixed scales confusing, and consistent direction or clear labeling was strongly recommended.	Breidenbach et al., 2021 [7]; Snyder et al., 2019 [31]
	Participants are divided on whether to reverse axes to enforce consistency; descriptive labels or shading are sometimes preferred alternatives.	Smith et al., 2016 [30]
	Inconsistent directionality and normed scoring are found to reduce accuracy of interpretation.	Tolbert et al., 2018 [32]
Labeling	Meaningful labeling of axes greatly improves interpretability. Descriptive y-axis labels (e.g., none/mild/moderate/severe) are favored over numeric-only axes by 79% of patients and 90% of clinicians. Labels also help mitigate confusion arising from directionality and unfamiliar statistical terms.	Smith et al., 2016 [30]; Fischer et al., 2020 [27]; Snyder et al., 2019 [31]
Normative comparison	Normed comparisons (e.g., to population averages) are helpful for clinicians but often confusing to patients, especially when not well explained.	Snyder et al., 2019 [31]; Brundage et al., 2015 [10]; Tolbert et al., 2018 [32]
	The reference group (e.g., general population vs. “patients like me”) should be clearly described. Personalized comparisons (same age, sex, diagnosis) are more meaningful to patients.	Hartzler et al., 2016 [33]
	Shaded projections of health trajectories were not always intuitive.	Fischer et al., 2020 [27]
Reference to treatment	Adding explicit reference to the treatment (e.g., knee replacement) within the visualizations helps contextualize PRO scores for patients, enhancing personal relevance.	Fischer et al., 2020 [27]
Time points	Flexible assessment intervals and accurate proportional spacing of time points along the x-axis are recommended but require further research.	Fischer et al., 2020 [27]; Snyder et al., 2019 [31]

## Results

### Literature and PRO tool review

The review of existing literature and available PRO tools with visualizations revealed an already broad knowledge base identifying key design dimensions relevant for visual presentation of health data. A summary of the findings is presented in Table 1. These thematic categories therefore guided the creation of the new designs evaluated in the user interviews.

### Interviews

The COREQ-checklist [36] was used to guide the reporting of this study (Online Resource 2). In total, eight patients and five healthcare providers from four different PRO B study centers were interviewed remotely via MS Teams. All interested participants were included apart from one who could not take part due to scheduling conflicts. Data saturation was discussed between LD and TP and considered to be reached. No follow-up interviews were conducted and participants did not provide feedback on the findings. The mean age of all participants was 48.3 years (SD = 7.8). Participant characteristics are listed in Table 2. All conducted interviews were recorded, transcribed and included in the qualitative content analysis.

**Table 2:** Participant characteristics

Participant ID	Role	PRO B study center (SC)	Age (years)
1	Study nurse	SC38	42
2	Study nurse	SC21	60
3	Study nurse	SC11	44
11	Breast cancer physician	SC13	n.a.
12	Breast cancer physician	SC21	33
**n**			**5**
**Mean (SD)**^a^			**44.8 (11.2)**
4	Patient	SC13	42
5	Patient	SC21	49
6	Patient	SC21	47
7	Patient	SC21	46
8	Patient	SC13	55
9	Patient	SC21	51
10	Patient	SC21	50
13	Patient	SC21	60
**n**			**8**
**Mean (SD)**			**50.0 (5.6)**
**n total**			**13**
**Mean (SD) total**			**48.3 (7.8)**

^a^SD = standard deviation, SC = study center

All graphical representations underlying the following interview results are presented in Online Resource 3.

We derived eight different thematic categories from the interviews, which reflect key design dimensions relevant for target users in clinical practice regarding the interpretation, emotional impact, and usability of PRO visualizations: graph labels, color scheme, background lines, alert representation, reference data, missing data, format and additional information, usage of the visualizations. Together, they provide a structured framework for translating user preferences and concerns into concrete design recommendations. Results were grouped by sub-themes within each category. We use quotes (e.g. #2) to illustrate these themes. Categories, themes and quotes are fully presented in Online Resource 4.

### Graph labels

**Fig. 2:**
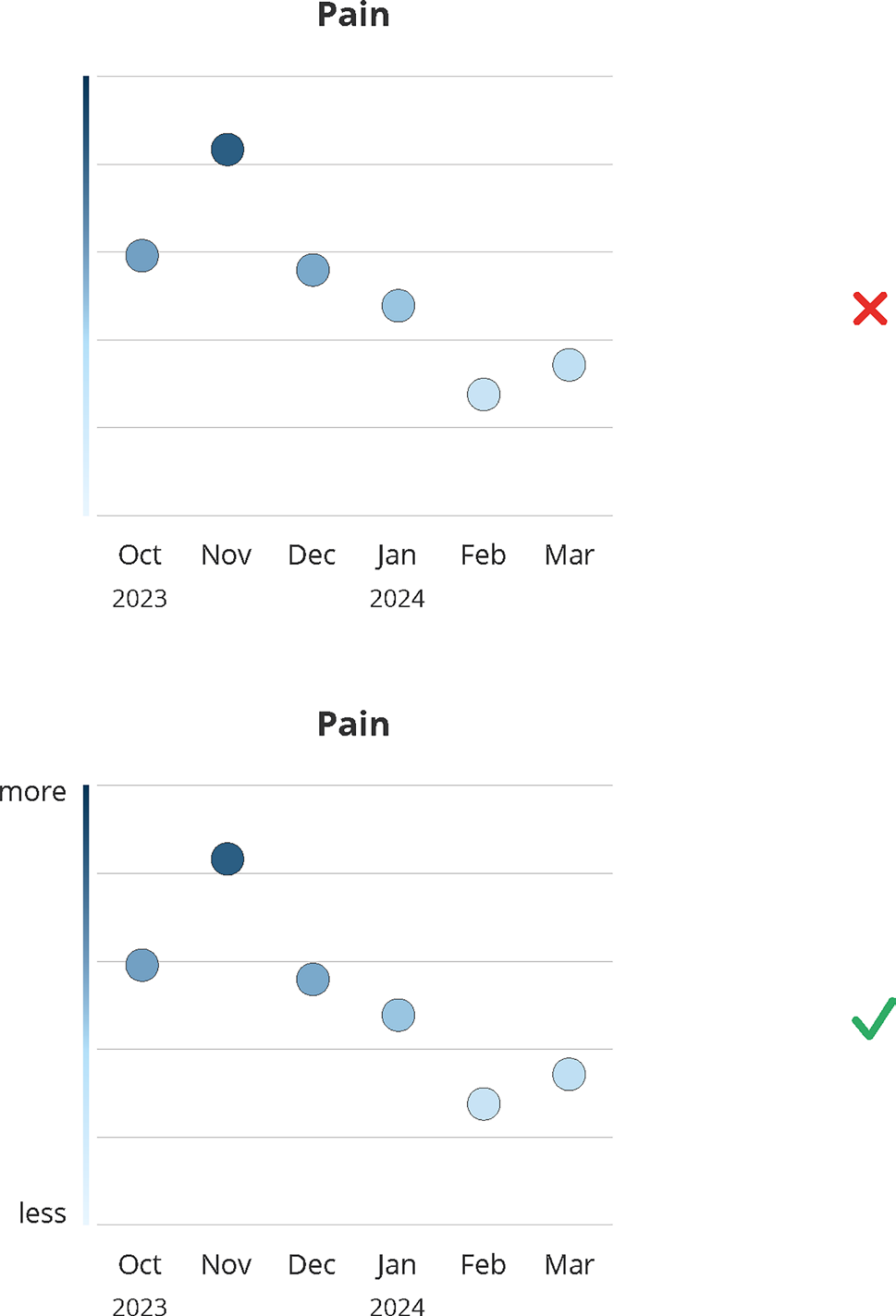
Design decision: graph labels. Green (check mark) indicates the final design, red (x) indicates the dismissed design; months abbreviations are listed on the x-axis

Participants emphasized the importance of clarity. While a color gradient indicating value expression along the y-axis was generally interpreted correctly, both patients and healthcare providers preferred clear labels to reduce ambiguity (#1–#5) (see Fig. 2), prioritizing clarity over aesthetics (#6, #7). Consensus held that designs should support general comprehensibility, as daily condition and cognitive function can fluctuate under cancer medication (#7–#9).

*It’s better that it’s understandable than that it looks nice in this case […] because, after all, you have good days and bad days too. (#7, patient)*

Labels particularly aid interpretation for patients unfamiliar with PRO domains or with abstract concepts in symptom and HRQoL questionnaires (e.g., “emotional functioning,” “role functioning”) (#10).

Arrows on the y-axis indicating direction (more/better at the top vs. less/worse at the bottom) were deemed unnecessary, as they did not improve comprehension and reduced clarity and balance (#11–#17).

Clear labels helped disambiguate directionality: Consistent presentation of high values at the top (i.e. good functioning, high symptom load) combined with a changing color gradient (good functioning = light color at the top, but high symptom load = dark color at the top), was easier and faster to understand when labels were presented at each end of the y-axis (#18, #19, #20). Even experienced providers valued labels for correct and rapid interpretation (#21, #22).

*I find this graph better because it clearly shows where there’s an increase, a deterioration, or an improvement. […] It’s definitely more advantageous because here it’s exactly the opposite of the deterioration and the improvement, which would otherwise be confusing. (#21, provider)*

However, one patient noted that the labels indicating ‘better’ outcomes could be discouraging, as the highest possible scores may feel unattainable given their current health status (#23).

**Design Recommendation**: Altogether, these findings emphasize to prioritize clear axis labeling over purely aesthetic considerations.

### Color scheme

**Fig. 3:**
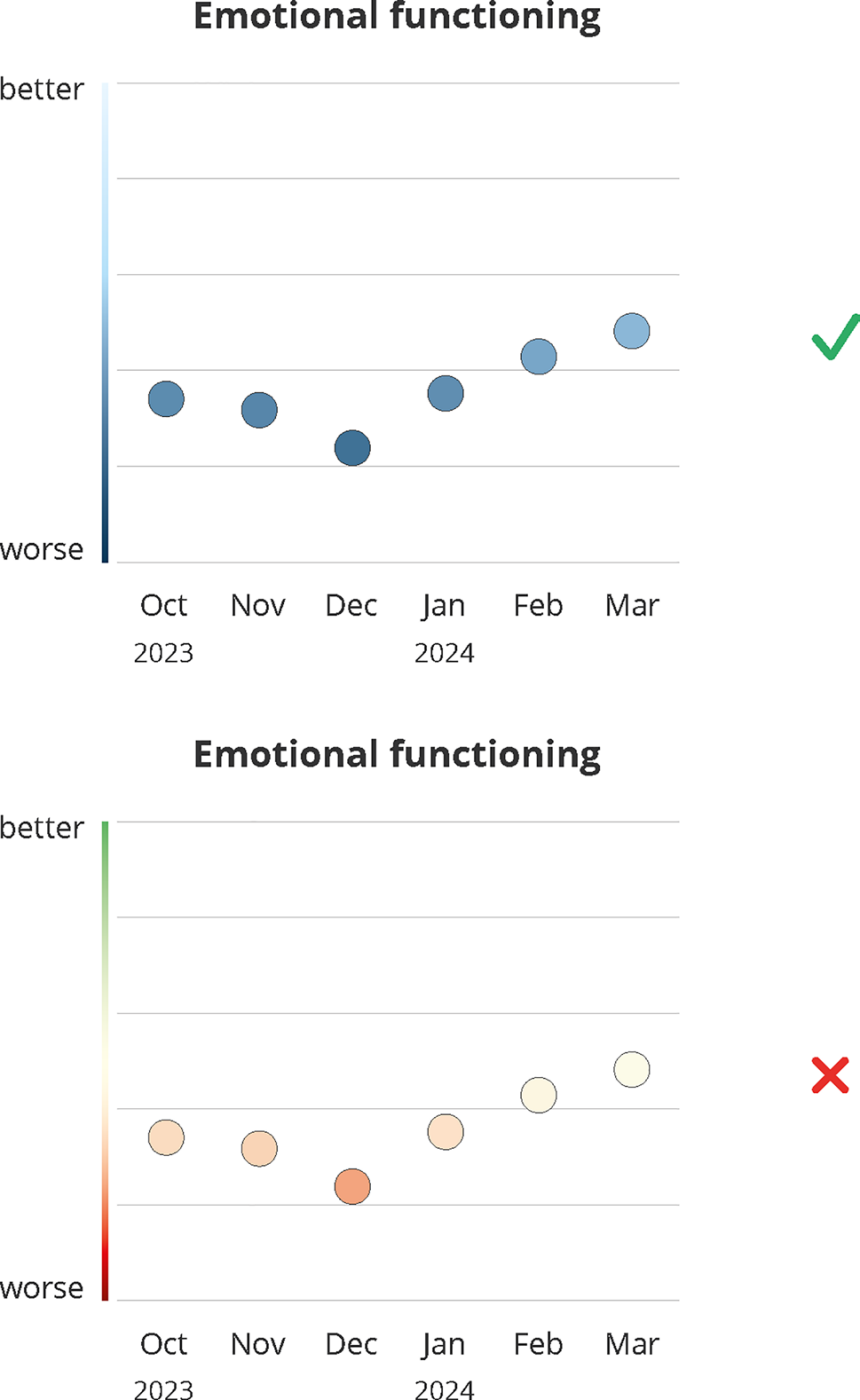
Design decision: color scheme. Green (check mark) indicates the final design, red (x) indicates the dismissed design; months abbreviations are listed on the x-axis

Participants were shown two color schemes (see Fig. 3): a traffic light (red–yellow–green) and a blue gradient (dark blue to light blue). Opinions were split: half of the patients favored the traffic light scheme for its familiar, clear signal effect (#24, #25), supported by providers, who noted red signals caution and green indicates balance or health (#26, #27). One provider noted that the traffic light scheme might be easier for patients to understand due to similarity with temperature scales (#28).

Some participants preferred the stronger color contrast of the traffic light scheme, particularly helpful for patients with reduced vision (#29, #30). Conversely, one provider argued that the traffic light spectrum may hinder differentiation for patients with protanomaly and/or deuteranomaly (#31). Patients who preferred the blue scale often associated red with blood and pain (#32):

*For me, red is an unpleasant phase. I associate it with pain, with blood. […] Yes, when you’re constantly getting blood drawn, when everyone’s always going for your veins. No, for me, it’s no longer the color of love, but rather the color of pain. (#32, patient)*

One patient noted that medical design guidelines often avoid red (#33). In contrast, one provider favored warm colors for emotional functioning data (#34), indicating color preferences may vary depend on the displayed domain.

**Design recommendation:** As a conclusion, designs should favor a neutral color palette to balance interpretability and emotional acceptability.

### Background lines

**Fig. 4:**
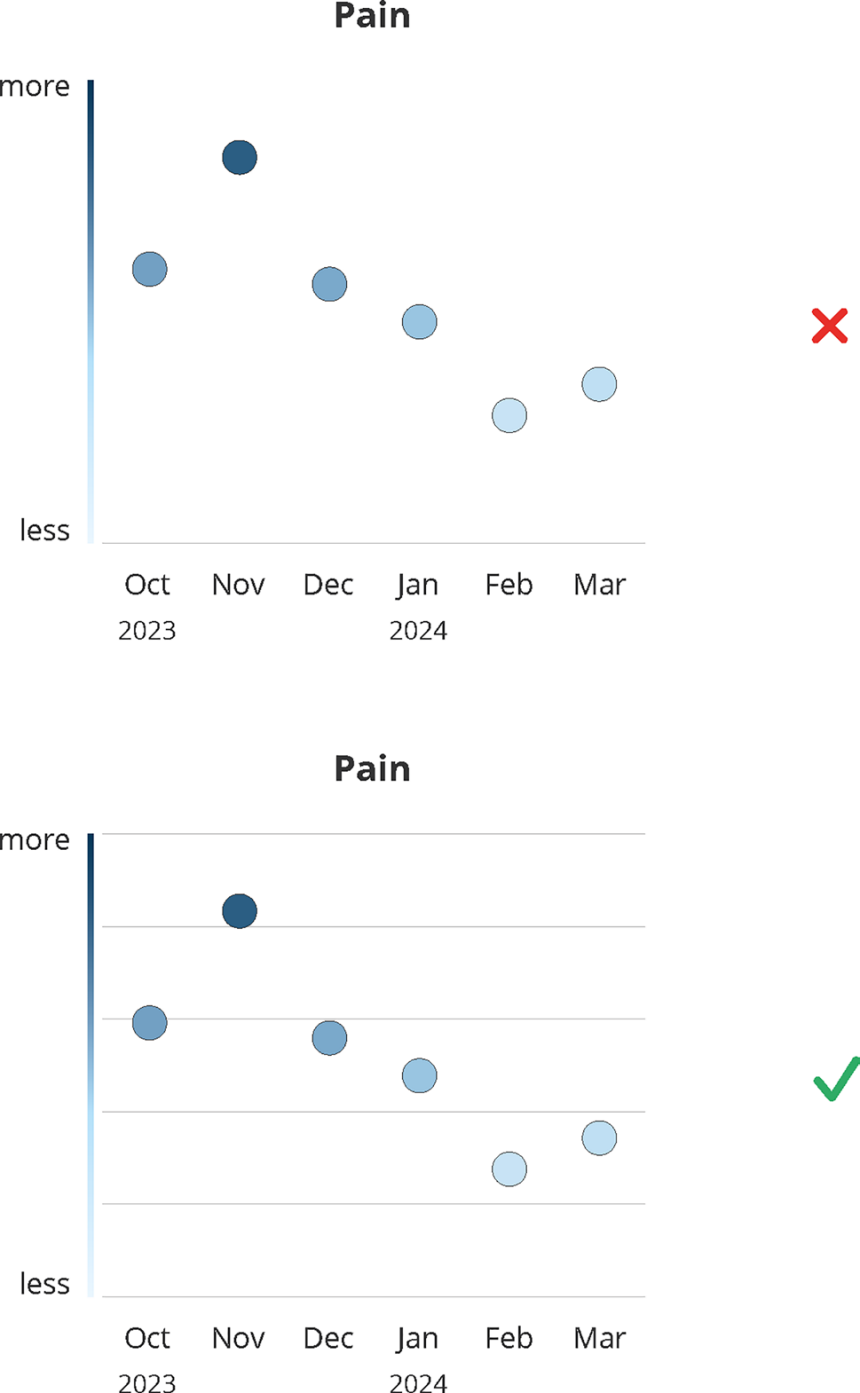
Design decision: background lines. Green (check mark) indicates the final design, red (x) indicates the dismissed design; months abbreviations are listed on the x-axis

Background lines were generally perceived as very subtle, with most participants not noticing them initially (#35–#43). Once recognized, they helped classify and rate data and enabled quicker comparison of individual data points (#44–#48) (see Fig. 4). Participants likened them to stave lines, preventing a sense of “getting lost” in the graph (#49–#51). Providers preferred lines that were noticeable yet subtle enough to avoid distracting from the data (#42, #43).

**Design recommendation:** Based on these responses, subtle background lines are recommended to support orientation without leading to distraction.

### Alert representation

**Fig. 5:**
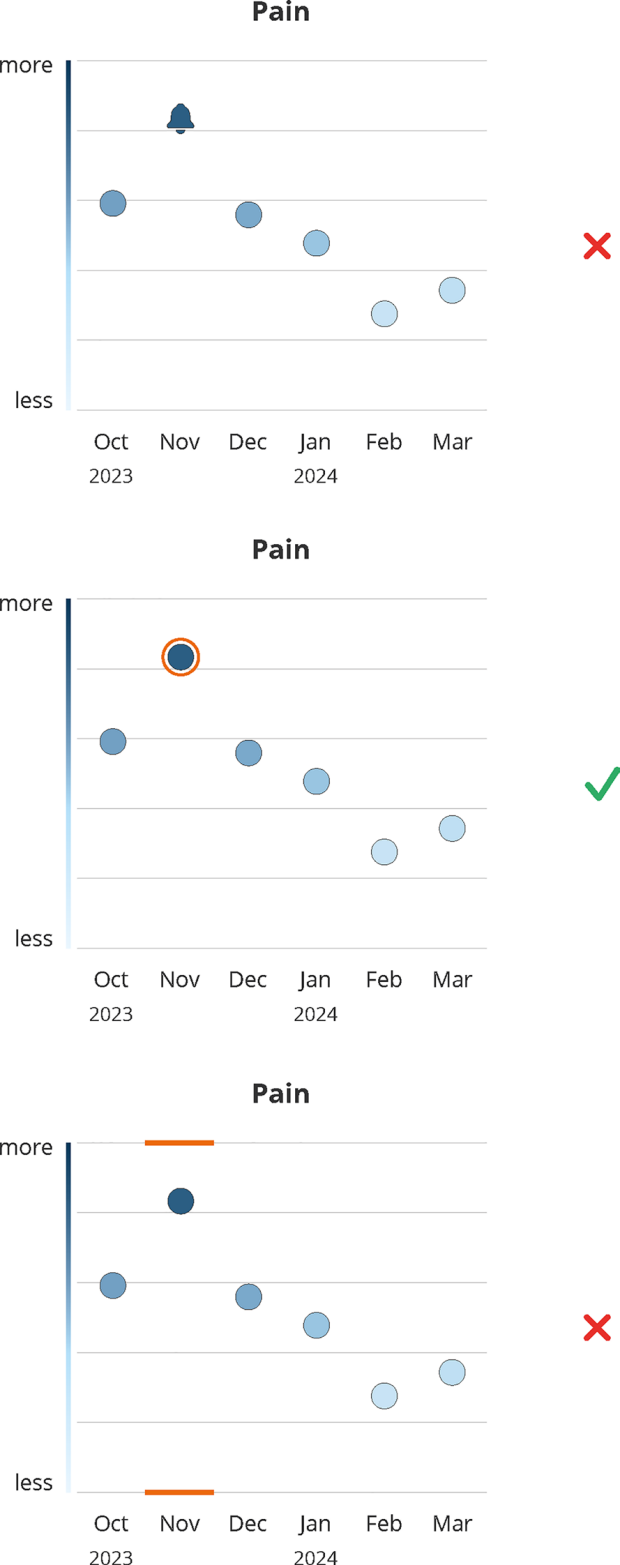
Design decision: alert representation. Green (check mark) indicates the final design, red (x) indicates the dismissed design; months abbreviations are listed on the x-axis

Patients and providers agreed that alert data should immediately draw attention (#52–#54). Alerts were best understood when represented simply, e.g. marking the data point without changing its shape (#55–#57) (see Fig. 5).

*The middle one, because it’s not too dominant but still clearly shows that the pain level was highest here. The bars […]—I can imagine that someone without experience might not be able to properly assess the bars, these lines. (#57, provider)*

Some preferred highlighting the corresponding time point of alert on the axis since it was more prominent (#58, #59) and helped identify the affected month (#60). However, this confused others (#61, #62), and providers noted it could distract from the data point (#63, #64). Some providers favored a symbolic alert representation such as conveying an acoustic nature (#65, #66), though there was no consensus on whether this improved comprehension (#67). Symbols risked implying interactivity, such as triggering an action by clicking (#68), and among patients, evoked symptom-specific associations (e.g., radiating or “screaming” pain) (#69, #70) that were not universally applicable. Preferences varied with single or multiple alerts. Consecutive alerts could provoke anxiety, so a calming, non-overloading design was preferred (#71–#75).

*Interestingly, I actually find the small ones the most appealing now, because then it’s not quite so bad. […] I’m more in favor of the one in the middle, because it’s such a good mix—you can see it, but it’s not like you think, “Oh my goodness, I’ve basically been living in a state of alarm from November to March”. (#74, patient)*

Most favored marking alert data points with a frame or circle, providing a clear, unambiguous reference without overwhelming the visualization (#73, #74).

**Design recommendation:** These insights led to the selection of a simple, minimally intrusive marking of alerted data points to ensure visibility while avoiding unnecessary emotional burden.

### Reference data

**Fig. 6:**
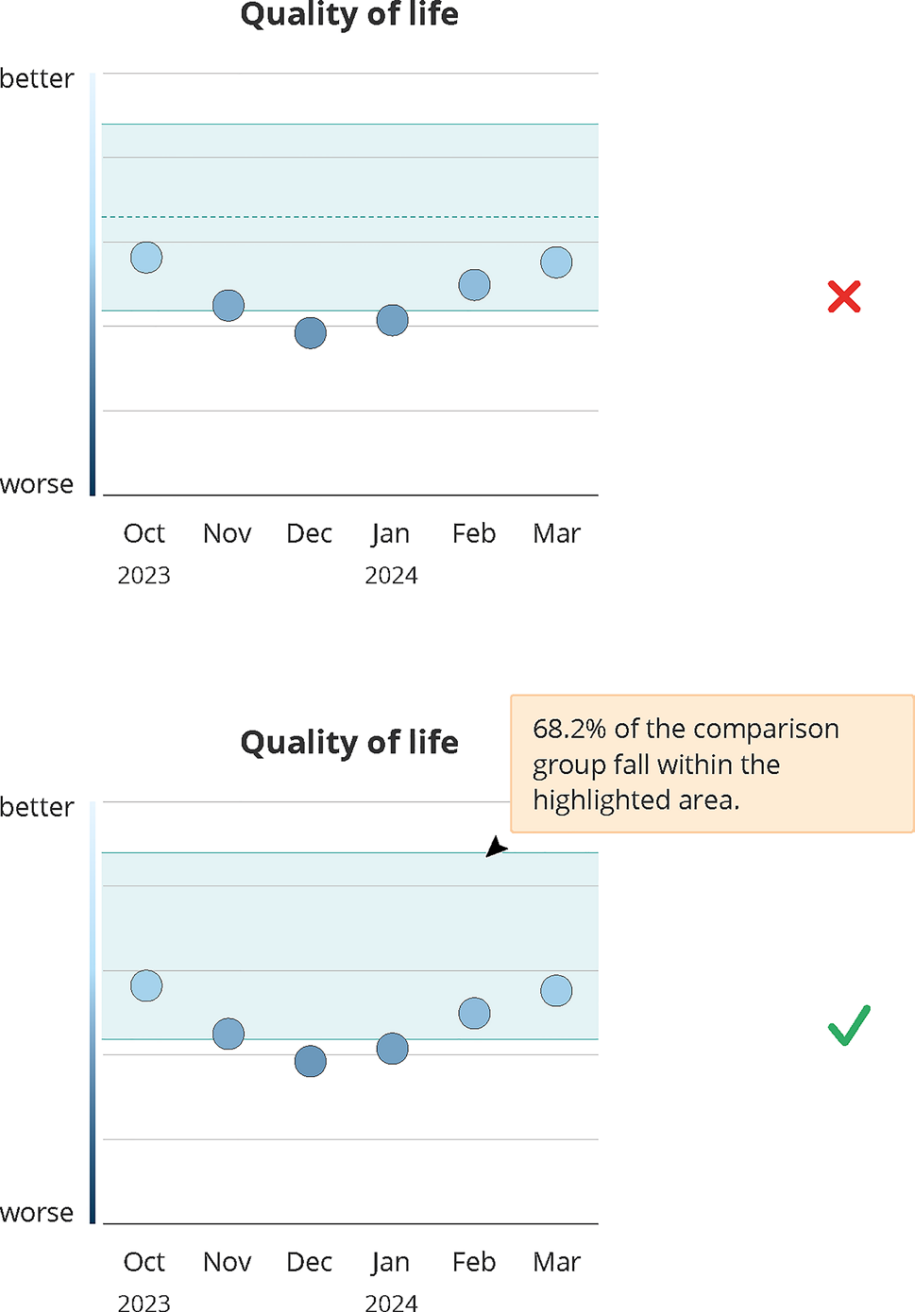
Design decision: reference data. Green (check mark) indicates the final design, red (x) indicates the dismissed design; “Quality of life” refers to the global health Status/quality of Life of the EORTC CAT core short form; months abbreviations are listed on the x-axis

Reference data, shown as a light blue background area, was generally understood as a tolerance range for acceptable values (#76–#79). The purpose of an additional mean-value line was unclear (#80–#82) and sometimes frustrating when suggesting values below a certain threshold, even within the tolerance range (#80, #81). The line prompted speculations even among providers (#83, #84) (see Fig. 6). Explanatory notes in the graph were appreciated, but participants requested a clear definition of the reference group (#85, #86), which was considered difficult to determine (#87).

*Rather, the question is, who is the comparison group? Are these exactly the patients in this and that age range, under this and that therapy? That is, of course, always extremely difficult in the individual situation, in the palliative situation, to define homogeneous groups as a comparison group. That would make me suspicious. (#87, provider)*

Reference data could evoke mixed emotions, including envy, pressure (#88, #89), reassurance and a sense of not being alone (#90, #91). Moreover, it could motivate discussions about treatment options (#92, #93).

*Well, on the other hand, it can also be an incentive to talk to my doctor about it, like why are they feeling better and what else can I do to make myself feel better? (#92, patient)*

Emotional reactions depended on current health or mood (#94, #95). Offering reference data on an optional basis was considered a potential solution (#96).

**Design recommendation:** Given the divergent emotional responses, reference data should be incorporated as an optional element, allowing users to control whether and when comparative information is displayed.

### Missing PRO data

**Fig. 7:**
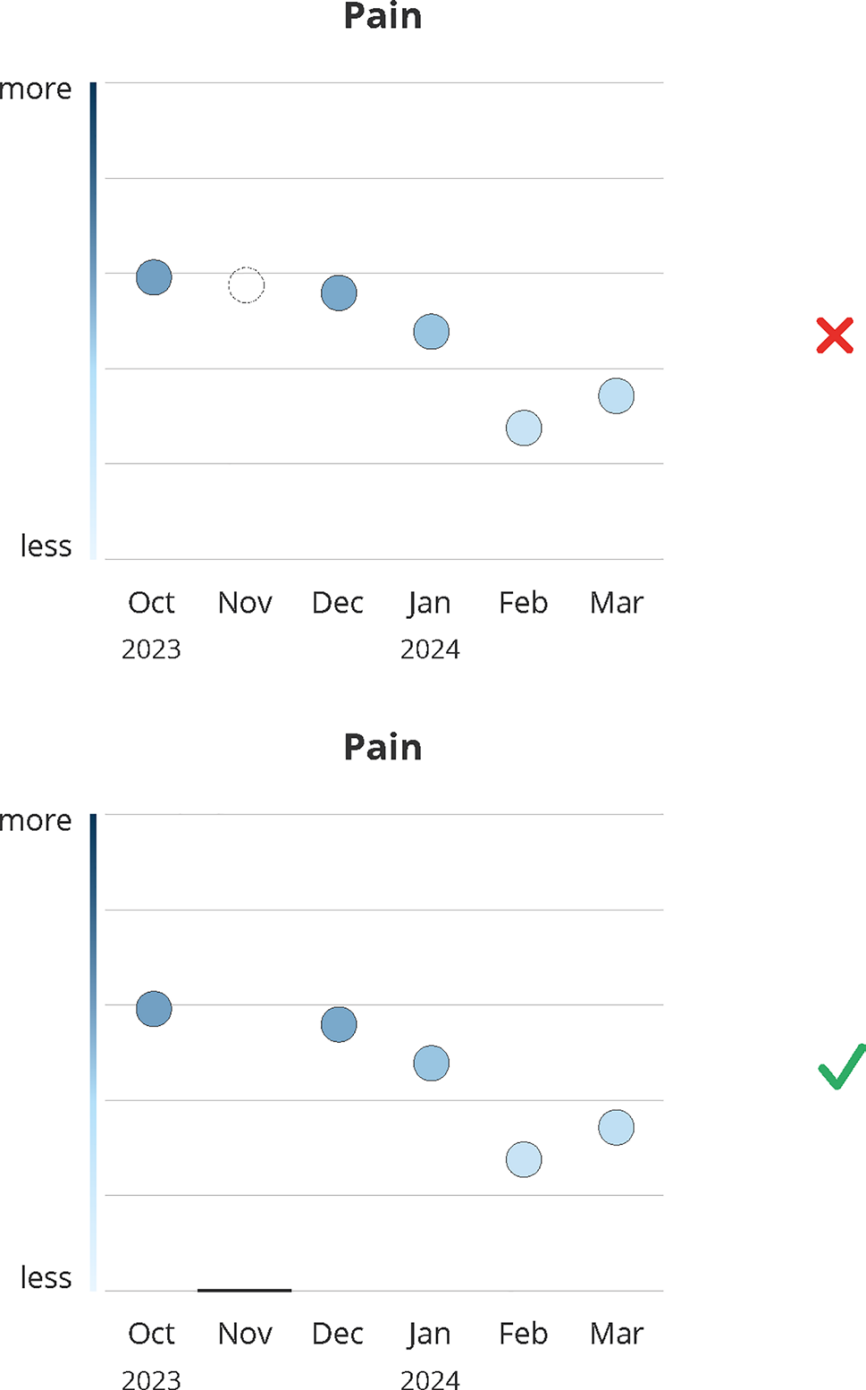
Design decision: missing data. Green (check mark) indicates the final design, red (x) indicates the dismissed design; months abbreviations are listed on the x-axis

Patients and providers criticized missing values as too subtle, making them hardly perceptible (#97–#101), which could lead to misinterpretation, e.g. assuming a printing error (#99). Patients preferred missing and non-missing points shown consistently as dots (#102, #103). Providers, stressed that dots should represent available data (#104), to avoid confusion with values outside the measurable range (#105) (see Fig. 7).

**Design recommendation:** These results underscored the need for a clearly distinguishable and consistently applied visual indicator for missing values to prevent misinterpretation.

### Format and additional information

Patients disagreed on whether a digital or a paper-based report would best meet their needs. Some preferred paper to bring to consultations (#106, #107), while others noted paperwork overload (#108). In digital formats, patients wanted personalization, e.g. zooming on data points (#109).

Providers favored digital tools that visualize PRO data and alerts and includes automated summaries of a patient’s health trends (#110, #111).

*I think digital is very, very good because it allows you to record progressions even better over a longer period of time. You don’t get all patients that way, but of course you get the younger ones, and younger people were certainly up to their mid-70s or so. For those over 80, the question is whether they want that. So I think digital is the right tool, it’s also the tool you can’t ignore. It’s the future. So I wouldn’t even ask the question of whether paper is better or worse. Instead, I’d rather work on the digital format, uh, still fine-tuning it. (#110, provider)*

Patients additionally requested brief evaluations of their data alongside graphs, providing an objective assessment based on medical expertise (#112), and the option to view additional medical information (vital signs, temperature curves) (#113). One patient suggested using visualizations to empower patients by highlighting positive developments (#114).

**Design recommendation:** Together, these preferences support the prioritization of a flexible digital format complemented by optional summary information and personalization features.

### Usage of the visualizations

Patients believed visualized PRO data could improve treatment by helping care teams to gain a better overall impression of their health and to detect critical situations earlier (#115, #116), facilitating the work of the care team (#116), improving communication between patients and providers, and providing emotional and psychological support. Knowing that a medically trained care team is monitoring their health was perceived as a “safety net” (#117). Visualizations were considered particularly helpful for introverted patients or those with a less close relationship to their care team, supporting discussion (#118–#120), active participation in treatment decisions (#121) and long-term reflection on health trends (#122, #123) in relation to specific events or interventions (#124–#126).

*Well, the first thing that comes to mind is that the doctor’s first question, “How are you?”, might then be superfluous, but of course it isn’t. Well, the doctor can of course, or I can say more specifically that I was already worse back then than this and that, for example, when the medication change took place. And that I’ve been feeling worse since then. I could then see that, become aware of it, and communicate it that way. (#126, patient)*

They could also aid documentation, e.g., for nursing care applications (#127). Providers valued visualizations as tool to guide consultations, facilitate communication, and foster shared decision-making (#128, #129), especially in long-term care of progressive diseases (#130).

Finally, some patients wanted the ability to choose when to view visualizations, opting to avoid them during stressful periods to prevent additional emotional strain (#131).

**Design recommendation:** Designs should position PRO visualizations as communication and reflection tools that support shared decision-making while respecting individual readiness to engage with the data.

## Discussion

This study aimed to develop user-centered visualizations of PRO data for use in the long-term care of patients with metastatic breast cancer. Embedded within the multicentric PRO B study, this work combined evidence from health data visualization with healthcare service design principles to ensure that the resulting visualizations met the real-world needs of both patients and providers. In doing so, our study responds to a persistent challenge in PRO implementation: despite evidence that PRO monitoring improves outcomes [2–4], adoption remains limited, often due to difficulties in interpreting and using PRO data [6, 7, 11].

Our qualitative findings highlight a critical need for intuitive, accessible, and emotionally sensitive PRO data presentation.

Clear labeling emerged as a key design feature, improving comprehension across diverse user groups and particularly benefiting individuals with limited familiarity with abstract PRO domains. Inconsistent directionality, commonly seen in existing tools, was identified as a major source of confusion, emphasizing the need for standardized scales and descriptive axis labels, consistent with earlier findings [7, 31].

In line with previous research [30], color use and alert formats proved more complex: while traffic-light schemes and prominent alerts can guide attention, they may also trigger negative emotions or risk misunderstandings. Neutral color palettes, simple markings, and user-customizable options help balance clarity with emotional sensitivity.

Similarly, reactions to reference data were mixed: some found it helpful for context and shared experience, while others felt discouraged by scores below the mean. Participants preferred optional, user-controlled displays – a need also raised elsewhere by providers [11] – highlighting the value of configurable digital formats.

Beyond facilitating interpretation, patients valued PRO visualizations for supporting self-reflection, long-term tracking, and informed discussions with clinicians. Providers saw them as tools for guiding conversations, facilitating shared decision-making, and enhancing care continuity. Together, these findings suggest that well-designed PRO visualizations function as boundary objects, bridging patient and provider perspectives and fostering more collaborative, patient-centered care.

By integrating the key design themes identified in this study, our results offer practical guidance for designers, developers, and healthcare teams. These insights are relevant not only for metastatic breast cancer care but also for broader PRO implementation, helping ensure that PRO tools are usable, interpretable, and emotionally acceptable across clinical settings.

## Limitations

Despite these insights, some limitations must be acknowledged. The sample size for qualitative interviews was modest and limited to participants from the PRO B study, potentially affecting the generalizability of results. Future studies should involve a broader and more diverse population to validate and refine the proposed visualization strategies. Moreover, user preferences may vary depending on disease stage, treatment phase, or emotional state – underscoring the importance of flexible, adaptive design.

## Conclusion

In conclusion, this study demonstrates the value of participatory design and qualitative evaluation in developing effective PRO visualizations. By grounding design decisions in user experiences, we can create tools that not only improve data comprehension but also enhance the active use of PRO data in clinical routine and thereby improve the overall quality of care in oncology.

## Data Availability

The experimental and qualitative data that support the findings of this study are included in this published article and its supplementary information files (Online Resources).

## Abbreviations

PRO: Patient-reported outcomes
HRQoL: Health-related quality of life
EORTC CAT: European Organization for Research and Treatment of Cancer (EORTC) Quality of Life Group Computerized Adaptive Testing (CAT) Core item bank

## Footnotes

1 The study name PRO B originated from a working title during study design and refers to Patient Reported Outcomes (PRO) in Breast Cancer (B).

2 PRO surveys comprised short forms from the European Organization for Research and Treatment of Cancer (EORTC) Quality of Life Group Computerized Adaptive Testing (CAT) Core item bank, which are scored as standardized T-scores (mean = 50, SD = 10) based on the general European population.

## References

[1] Valderas JM, Kotzeva A, Espallargues M, Guyatt G, Ferrans CE, Halyard MY et al (2008) The impact of measuring patient-reported outcomes in clinical practice: a systematic review of the literature. Qual Life Res 17(2):179–193 18175207 10.1007/s11136-007-9295-0

[2] Basch E, Deal AM, Kris MG, Scher HI, Hudis CA, Sabbatini P et al (2016) Symptom monitoring with patient-reported outcomes during routine cancer treatment: a randomized controlled trial. J Clin Oncol 34(6):557–565 26644527 10.1200/JCO.2015.63.0830 PMC4872028

[3] Denis F, Basch E, Septans AL, Bennouna J, Urban T, Dueck AC et al (2019) Two-year survival comparing Web-based symptom monitoring vs routine surveillance following treatment for lung cancer. JAMA 321(3):306–307 30667494 10.1001/jama.2018.18085 PMC6439676

[4] Calvert M, Blazeby J, Altman DG, Revicki DA, Moher D, Brundage MD et al (2013) Reporting of patient-reported outcomes in randomized trials: the CONSORT PRO extension. JAMA 309(8):814–822 23443445 10.1001/jama.2013.879

[5] Absolom K, Warrington L, Hudson E, Hewison J, Morris C, Holch P et al (2021) Phase III randomized controlled trial of eRAPID: eHealth intervention during chemotherapy. J Clin Oncol 39(7):734–747 33417506 10.1200/JCO.20.02015

[6] Foster A, Croot L, Brazier J, Harris J, O’Cathain A (2018) The facilitators and barriers to implementing patient reported outcome measures in organisations delivering health related services: a systematic review of reviews. J Patient Rep Outcomes 2:46 30363333 10.1186/s41687-018-0072-3 PMC6170512

[7] Breidenbach C, Kowalski C, Wesselmann S, Sibert NT (2021) Could existing infrastructure for using patient-reported outcomes as quality measures also be used for individual care in patients with colorectal cancer? BMC Health Serv Res 21(1):448 33975586 10.1186/s12913-021-06457-6 PMC8111716

[8] Karsten MM, Speiser D, Hartmann C, Zeuschner N, Lippold K, Kiver V et al (2018) Web-based patient-reported outcomes using the International consortium for health outcome measurement dataset in a major German University Hospital: observational study. JMIR Cancer 4(2):e11373 30573450 10.2196/11373 PMC6320408

[9] McNair AG, Brookes ST, Davis CR, Argyropoulos M, Blazeby JM (2010) Communicating the results of randomized clinical trials: do patients understand multidimensional patient-reported outcomes? J Clin Oncol 28(5):738–743 20065187 10.1200/JCO.2009.23.9111

[10] Brundage MD, Smith KC, Little EA, Bantug ET, Snyder CF, Prodpsa B (2015) Communicating patient-reported outcome scores using graphic formats: results from a mixed-methods evaluation. Qual Life Res 24(10):2457–2472 26012839 10.1007/s11136-015-0974-y PMC4891942

[11] Albers EAC, Fraterman I, Walraven I, Wilthagen E, Schagen SB, van der Ploeg IM et al (2022) Visualization formats of patient-reported outcome measures in clinical practice: a systematic review about preferences and interpretation accuracy. J Patient Rep Outcomes 6(1):18 35239055 10.1186/s41687-022-00424-3 PMC8894516

[12] McCaffery KJ, Holmes-Rovner M, Smith SK, Rovner D, Nutbeam D, Clayman ML et al (2013) Addressing health literacy in patient decision aids. BMC Med Inf Decis Mak 13(Suppl 2):S10 10.1186/1472-6947-13-S2-S10 PMC4042520 24624970

[13] Sorensen K, Van den Broucke S, Fullam J, Doyle G, Pelikan J, Slonska Z et al (2012) Health literacy and public health: a systematic review and integration of definitions and models. BMC Public Health 12:80 22276600 10.1186/1471-2458-12-80 PMC3292515

[14] Houts PS, Doak CC, Doak LG, Loscalzo MJ (2006) The role of pictures in improving health communication: a review of research on attention, comprehension, recall, and adherence. Patient Educ Couns 61(2):173–190 16122896 10.1016/j.pec.2005.05.004

[15] Zikmund-Fisher BJ, Scherer AM, Witteman HO, Solomon JB, Exe NL, Tarini BA et al (2017) Graphics help patients distinguish between urgent and non-urgent deviations in laboratory test results. J Am Med Inf Assoc 24(3):520–528 10.1093/jamia/ocw169 PMC5565988 28040686

[16] Garcia-Retamero R, Cokely ET (2017) Designing visual aids that promote risk literacy: a systematic review of health research and evidence-based design heuristics. Hum Factors 59(4):582–627 28192674 10.1177/0018720817690634

[17] Mayer RE (2005) Cognitive theory of Multimedia Learning. In: Mayer R (ed) The Cambridge handbook of multimedia learning. Cambridge handbooks in psychology. Cambridge University Press, Cambridge, pp 31–48

[18] Franconeri SL, Padilla LM, Shah P, Zacks JM, Hullman J (2021) The science of visual data communication: what works. Psychol Sci Public Interest 22(3):110–161 34907835 10.1177/15291006211051956

[19] Antunes B, Harding R, Higginson IJ, Euroimpact (2014) Implementing patient-reported outcome measures in palliative care clinical practice: a systematic review of facilitators and barriers. Palliat Med 28(2):158–175 23801463 10.1177/0269216313491619

[20] Bantug ET, Coles T, Smith KC, Snyder CF, Rouette J, Brundage MD et al (2016) Graphical displays of patient-reported outcomes (PRO) for use in clinical practice: what makes a pro picture worth a thousand words? Patient Educ Couns 99(4):483–490 26603445 10.1016/j.pec.2015.10.027

[21] Debbeler LJ, Wahl DR, Villinger K, Renner B (2020) Die Bedeutung der Gesundheitskommunikation in der Prävention und Gesundheitsförderung. In: Tiemann M, Mohokum M (eds) Prävention und Gesundheitsförderung. Springer Reference Pflege - Therapie - Gesundheit. Springer Berlin Heidelberg, Berlin, Heidelberg, pp 1–11

[22] Karsten MM, Kuhn F, Pross T, Blohmer JU, Hage AM, Fischer F et al (2021) PRO B: evaluating the effect of an alarm-based patient-reported outcome monitoring compared with usual care in metastatic breast cancer patients-study protocol for a randomised controlled trial. Trials 22(1):666 34583744 10.1186/s13063-021-05642-6 PMC8479993

[23] Gebert P, Karsten MM, Hage AM, Dordevic AD, Grittner U (2024) Statistical analysis plan for the PRO B study: open-label, superiority randomised controlled trial of alarm-based patient-reported outcome monitoring in patients with metastatic breast cancer. Trials 25(1) 10.1186/s13063-024-08025-9 PMC10918931 38448904

[24] Clack LA, Ellison RL (2019) Innovation in Service design Thinking. In: Pfannstiel MA, Rasche C (eds) Service design and service thinking in healthcare and hospital management. Springer International Publishing, Cham, pp 85–92

[25] Stickdorn M, Lawrence A, Hormess M, Schneider J, ORfH E (2017) This is service design doing: applying service design thinking in the real world: a practitioner’s handbook. O’Reilly Media

[26] Kuckartz U (2014) Qualitative text analysis: a guide to methods, practice & using software. SAGE

[27] Fischer KI, De Faoite D, Rose M (2020) Patient-reported outcomes feedback report for knee arthroplasty patients should present selective information in a simple design - findings of a qualitative study. J Patient Rep Outcomes 4(1):6 31965364 10.1186/s41687-020-0173-7 PMC6973599

[28] Gilbert A, Sebag-Montefiore D, Davidson S, Velikova G (2015) Use of patient-reported outcomes to measure symptoms and health related quality of life in the clinic. Gynecol Oncol 136(3):429–439 25448486 10.1016/j.ygyno.2014.11.071

[29] Snyder CF, Smith KC, Bantug ET, Tolbert EE, Blackford AL, Brundage MD et al (2017) What do these scores mean? Presenting patient-reported outcomes data to patients and clinicians to improve interpretability. Cancer 123(10):1848–1859 28085201 10.1002/cncr.30530 PMC5419857

[30] Smith KC, Brundage MD, Tolbert E, Little EA, Bantug ET, Snyder CF et al (2016) Engaging stakeholders to improve presentation of patient-reported outcomes data in clinical practice. Support Care Cancer 24(10):4149–4157 27165054 10.1007/s00520-016-3240-0

[31] Snyder C, Smith K, Holzner B, Rivera YM, Bantug E, Brundage M et al (2019) Making a picture worth a thousand numbers: recommendations for graphically displaying patient-reported outcomes data. Qual Life Res 28(2):345–356 30306533 10.1007/s11136-018-2020-3 PMC6363861

[32] Tolbert E, Brundage M, Bantug E, Blackford AL, Smith K, Snyder C et al (2018) Picture this: presenting longitudinal patient-reported outcome research study results to patients. Med Decis Mak 38(8):994–1005 10.1177/0272989X18791177 PMC6221949 30132393

[33] Hartzler AL, Izard JP, Dalkin BL, Mikles SP, Gore JL (2016) Design and feasibility of integrating personalized PRO dashboards into prostate cancer care. J Am Med Inf Assoc 23(1):38–47 10.1093/jamia/ocv101 PMC5009933 26260247

[34] Hennink MM, Kaiser BN, Marconi VC (2017) Code saturation versus meaning saturation: how many interviews are enough? Qual Health Res 27(4):591–608 27670770 10.1177/1049732316665344 PMC9359070

[35] Saunders B, Sim J, Kingstone T, Baker S, Waterfield J, Bartlam B et al (2018) Saturation in qualitative research: exploring its conceptualization and operationalization. Qual Quant 52(4):1893–1907 29937585 10.1007/s11135-017-0574-8 PMC5993836

[36] Tong A, Sainsbury P, Craig J (2007) Consolidated criteria for reporting qualitative research (COREQ): a 32-item checklist for interviews and focus groups. Int J Qual Health Care 19(6):349–357 17872937 10.1093/intqhc/mzm042

